# Development and Implementation of Culturally Tailored Offline Mobile Health Surveys

**DOI:** 10.2196/publichealth.5408

**Published:** 2016-06-02

**Authors:** Scott McIntosh, José Pérez-Ramos, Margaret M Demment, Carmen Vélez Vega, Esteban Avendaño, Deborah J Ossip, Timothy D Dye

**Affiliations:** ^1^ School of Medicine & Dentistry Department of Public Health Sciences University of Rochester Rochester, NY United States; ^2^ School of Medicine & Dentistry Clinical and Translational Science Institute University of Rochester Rochester, NY United States; ^3^ Recinto de Ciencias Médicas Departamento de Ciencias Sociales Universidad de Puerto Rico San Juan Puerto Rico; ^4^ Universidad de Ciencias Médicas San José Costa Rica; ^5^ School of Medicine & Dentistry Department of Obstetrics & Gynecology University of Rochester Rochester, NY United States

**Keywords:** mobile health, survey research, ethical review

## Abstract

**Background:**

In low and middle income countries (LMICs), and other areas with low resources and unreliable access to the Internet, understanding the emerging best practices for the implementation of new mobile health (mHealth) technologies is needed for efficient and secure data management and for informing public health researchers. Innovations in mHealth technology can improve on previous methods, and dissemination of project development details and lessons learned during implementation are needed to provide lessons learned to stakeholders in both the United States and LMIC settings.

**Objective:**

The aims of this paper are to share implementation strategies and lessons learned from the development and implementation stages of two survey research projects using offline mobile technology, and to inform and prepare public health researchers and practitioners to implement new mobile technologies in survey research projects in LMICs.

**Methods:**

In 2015, two survey research projects were developed and piloted in Puerto Rico and pre-tested in Costa Rica to collect face-to-face data, get formative evaluation feedback, and to test the feasibility of an offline mobile data collection process. Fieldwork in each setting involved survey development, back translation with cultural tailoring, ethical review and approvals, data collector training, and piloting survey implementation on mobile tablets.

**Results:**

Critical processes and workflows for survey research projects in low resource settings were identified and implemented. This included developing a secure mobile data platform tailored to each survey, establishing user accessibility, and training and eliciting feedback from data collectors and on-site LMIC project partners.

**Conclusions:**

Formative and process evaluation strategies are necessary and useful for the development and implementation of survey research projects using emerging mHealth technologies in LMICs and other low resource settings. Lessons learned include: (1) plan institutional review board (IRB) approvals in multiple countries carefully to allow for development, implementation, and feedback, (2) in addition to testing the content of survey instruments, allow time and consideration for testing the use of novel mHealth technology (hardware and software), (3) incorporate training for and feedback from project staff, LMIC partner staff, and research participants, and (4) change methods accordingly, including content, as mHealth technology usage influences and is influenced by the content and structure of the survey instrument. Lessons learned from early phases of LMIC research projects using emerging mHealth technologies are critical for informing subsequent research methods and study designs.

## Introduction

Electronic methods for capturing survey data have proven feasible [1] and efficient [2,3] in locales with the technical infrastructure to support their implementation [2,4]. In settings where the telecommunications and technology infrastructure may be uncertain or lacking and where Internet penetration may be lower, electronic means for capturing survey data is of limited utility [4]. Maximizing the benefits and efficiencies of electronic data capture in survey research in settings with limited technological infrastructure requires the decoupling of the means of collection (eg, mobile device, laptop computer) from the mechanism of transmission and storage of survey data (eg, via the Internet). Using a store-and-forward approach to data collection in resource-limited settings may offer an effective approach to survey research by achieving efficiency gains through mobile device-based data capture delayed synchronization of data [5-7] when access to the Internet becomes available.

Public health lessons from resource-challenged areas of the world increasingly inform practice in other areas, including higher-resourced communities. Knowledge translation from research into practice has long challenged public health [8]. Evidence-based interventions exist to reduce chronic disease mortality substantially by addressing common lifestyle and behavioral risks [9], but suffer from lack of implementation [10]. This gap between knowledge and practice – the “know-do” gap – is more pronounced in communities of need throughout the world [11]. Better understanding of local social and cultural circumstances may help reduce the know-do gap [12] and increasingly successful implementation of existing knowledge to reduce chronic illness requires community-based approaches [13]. Further, novel ways to achieve better engagement in communities with disparities frequently arise from global settings [14] that have demonstrated creative problem-solving when challenged with a wide range of barriers [15]. Such learning can directly benefit communities of need nested within wealthier nations [12], in part by recognizing that community circumstances warrant creativity in blending best-practice with local culture [16] to reduce preventable chronic disease mortality [17]. Creating, nurturing and supporting researcher-community partnerships are an essential component of this effective translation [18], though progress in bridging the know-do gap is improbable without the appropriate involvement of enabling technology and informatics support [19].

The Centers for Disease Control and Prevention (CDC), through its Prevention Research Centers (PRC) Program, established a thematic network, the Global and Territorial Health Research Network (Global Network) to conduct, share, and translate innovative chronic disease prevention research in low-resource settings. In the context of the Global Network, the University of Rochester partnered with the University of Puerto Rico to test the feasibility of an electronically-implemented survey of community attitudes toward participation in genetic research (Survey 1), a scientific area of increased importance [20]. The investigators used the same approaches for a separate electronic health (eHealth) survey (for new mothers) being developed with research partners in Costa Rica and the Dominican Republic (Survey 2). This report focuses on the field-testing of electronic surveys with offline, store-and-forward data capturing using Research Electronic Data Capture (REDCap), a widely-used data capture application developed for large-scale research projects [21]. Procedures, infrastructure concerns, and methodologies have been adapted from previous work and lessons learned [22-35] to meet the needs for maximum flexibility and usability in low-resource settings. Procedures for successful project implementation include ethical review, survey development, data collector training, pre-testing, pilot testing, and data management. In two low and middle income countries (LMIC) settings: Puerto Rico (Survey 1) and Costa Rica (Survey 2). The surveys were in initial development (pre-testing) and/or pilot testing phases and although the topical areas were different, the initiatives involved overlapping teams and lessons learned will continue to be useful across future initiatives with LMICs.

## Methods

### Ethical Review Process

Obtaining institutional review board (IRB) approval from all participating groups raises certain challenges [28]. For all research funded by US federal agencies and institutions, participating institutions in the United States and each participating country must have a Federal Wide Assurance (FWA) and approval from an Office for Human Research Protections (OHRP) registered IRB. The FWA provides a commitment by the institution to follow standard ethical guidelines for human subjects research (eg, the Belmont Report, the Declaration of Helsinki, or similar codes). To obtain an FWA, institutions must have a designated IRB registered with OHRP. A database of institutions with FWAs and IRBs that meet US human subjects’ protections guidelines for research is maintained by OHRP [36]. Each participating country outside of the United States may have its own required IRB for in-country research, which may not be approved by the US OHRP [28]. Thus, reviews by multiple IRBs may be required. Coordination of timing of reviews is critical to ensure that all are completed prior to study initiation. In addition, some IRBs may require approval by IRBs in the other participating countries prior to providing local approval, so it is critical for investigators to be aware of such local regulations and communicate documentation of approvals to each IRB within the required time frame [22,24,37,38].

Finally, investigators need to be aware of culturally-specific variations in what are acceptable research practices to produce a final protocol that meets regulatory requirements of each country. For example, in our prior work in the Dominican Republic, paying subjects for completing surveys, a practice that is acceptable by US IRB standards was considered coercive by Dominican Republic IRB standards, though provision of a small thank you gift at the end of the survey was acceptable with the caveat that participants not be informed of the gift until after completing the survey. In addition, in partner Dominican Republic communities, providing written consent for participation was culturally unacceptable as it raised concerns about contractual requirements. Thus, investigators worked with all IRBs to develop an acceptable verbal consent procedure with documentation of consent provided by data collectors [22,24,27,28]. Understanding IRB requirements of all participating sites is critical to successful global partnerships for research [37].

The IRB process at the University of Puerto Rico (UPR/MSC) was another source of lessons learned. The initial request for IRB approval was submitted for the online/offline versions of the survey to be administered by means of iPads and facilitated by a member of the research team. Once the IRB approval was ready (see below), the team proceeded to collect data. Once data collection began, the team faced the challenge of implementing offline collection, as the study sites were in remote areas in Puerto Rico with limited access to an Internet connection. Not being able to use the printed, hard copy as an alternative to the electronic version was a setback that required an amendment to the original protocol and changes to the data collection schedule. The lessons learned here – to gain initial IRB approval for both electronic and paper versions of survey data collection - can help to avoid further delays in the study plan.

### Participants

#### Survey 1

Participants in Survey 1 consisted of a convenience sample of 32 Puerto Rican residents who were engaging with their medical health care system in one of three Federally Qualified Health Centers (FQHCs) in rural areas of Northern Puerto Rico over a 2-week period. Potential participants were approached by project data collectors (see below) and, if agreeable, were provided an information letter about the purpose of the survey. Implementing the survey consisted of a face-to-face interview, using a digital tablet to enter responses.

#### Survey 2

In the development phase of the study prior to an actual pilot implementation of Survey 2, qualitatively pre-testing all the sections and items of the developing survey was accomplished with a convenience sample of 16 trainees who were participating in an National Institute of Health (NIH) funded eHealth training initiative, and who will later be data collectors using Survey 2 and offline data collection. This sample was mostly composed of highly educated persons (eg, health providers, technology experts) who will, later in the parent study, be responsible for administering the final version of Survey 2 in their own countries (Costa Rica, Honduras, and Dominican Republic). Feedback was obtained on content, wording, and device navigation (ie, using the REDCap mobile survey on a digital tablet).

### Data Collectors

#### Survey 1

For the field work with Survey 1 in Puerto Rico, data collectors were six Puerto Rican-based staff who were participating in the Puerto Rico Testsite for Exploring Contamination Threats (PROTECT), a research project that follows a cohort of 1800 pregnant women in the northern Karst area of Puerto Rico and their exposure to environmental agents [39,40]. Biological samples for PROTECT, an IRB-approved study, include examination of the DNA for the PAX gene and for future genetic testing.

The PROTECT staff that participated in the field work with Survey 1 included one registered nurse and fieldwork coordinator with ample experience in research, one master’s degree nutritionist, a doctoral student with a master's in public health (MPH) degree and experience in field research, and the co-principal director for the PROTECT Community Engagement Core with a doctorate of philosophy (PhD) in policy analysis and research. The staff were all previously familiar with REDCap, although they had not yet used the mobile version.

Training activities included observation and a 3-hour workshop to become familiar with both the content of the survey as well as its online and offline implementation via the mobile REDCap protocol. Each staff was given a tablet and assigned a unique username. All staff members participating in the project completed human subject research protection training from the Collaborative Institutional Training Initiative (CITI) prior to the collection of any data.

#### Survey 2

Qualitative data collection for the pre-testing phase of Survey 2 was conducted by a senior US research project staff (behavioral scientist), who interactively presented a draft of the draft survey as part of a didactic module during a week-long short course in Costa Rica (see below) on online/offline digital data collection. During the module and in subsequent meetings, senior project staff documented qualitative feedback via field notes and confirmed common themes and item improvement ideas. At the same time, another senior project team member provided live support and feedback from the United States, including real-time problem-solving that led to lessons learned (eg, problems with offline/online synchronization, log-in credentials, and other functionality).

### Instruments

#### Survey 1: From Mechanical Turk to Offline

The present fieldwork began with the development of a Spanish-language version of a survey initially available online at Mechanical Turk (mTurk), Amazon's crowdsourcing website [41-43]. The initial survey was assembled primarily from items validated in previous surveys. Reliability and validity have not yet been established, but it is currently in use in several of our initiatives and will be examined for reliability metrics in subsequent phases of project development. The Internet as a medium and, more recently, the use of “crowdsourcing” as a strategy, has greatly expanded the potential for low-cost timely survey research initiatives. Crowdsourcing is the paid (or often unpaid) “recruitment of an independent global workforce for the objective of working on a specifically defined task or set of tasks” [44]. Crowdsourcing is increasingly recognized as a legitimate strategy to engage with research subjects [45-47].

#### Spanish Back Translation Process

In order to create an equivalent, as well as culturally appropriate Spanish language version of the initial English version, it was first translated into Spanish, and then back translated into English using the Brislin method [48]. The team then compared the back-translated version with the original to identify problematic translations. These problematic translations were then examined by two native Puerto Rican speakers and edits were provided. Finally, a native Spanish speaker did a final edit of the entire survey before it was pre-tested. The Spanish version was pre-tested during the back translation process by six bilingual investigators from the United States and Puerto Rico for readability, skip patterns, formatting, and content. Committed partners and an organized and systematic approach facilitated the translation process. A barrier to the process was time to complete translation given that people were volunteering their time.

#### Research Electronic Data Capture (REDCap)

For the present field work, surveys were developed and implemented using the mobile (offline) application of REDCap, a software toolset and workflow methodology for electronic collection and management of research and clinical trial data [21]. The secure, Web-based application (whether online or offline) provides an intuitive interface for users to enter data and real-time validation rules (with automated data type and range checks) at the time of data entry. REDCap offers easy data manipulation with audit trails and functionality for reporting, monitoring and querying patient records, as well as an automated export mechanism to common statistical packages (SPSS, SAS, Stata, R/S-Plus).

The NIH-funded Clinical and Translational Science Institute’s (CTSI’s) informatics core, a unit of the University of Rochester's School of Medicine & Dentistry Academic Information Technology (AIT) Group, serves as a central facilitator for data processing and management. REDCap data collection projects rely on a thorough study-specific data dictionary defined in an iterative self-documenting process by all members of the research team, with planning assistance from the AIT-CTSI informatics core. The iterative development and testing process result in a well-planned data collection strategy for individual studies.

REDCap servers are housed in a local data center at the University of Rochester and all Web-based information transmission is encrypted. REDCap was developed in a manner consistent with Health Insurance Portability and Accountability Act (HIPAA) security requirements and is recommended to University of Rochester researchers by the University of Rochester Medical Center (URMC) research privacy officer and office for Human Subject Protection.

#### REDCap Mobile for Offline Data Collection

For the purpose of this fieldwork the REDCap application for offline data collection was used, because many surveillance sites will be remote and will have limited Internet access. Mobile offline data collection allows for real-time data collection and storage. Data are later “synced” or uploaded to an online server and added to the primary database.

Although software programs and hardware devices were not prospectively compared and tested with our LMIC project partners, we employed lessons learned from our previous work regarding reach and engagement considerations of eHealth strategies [49,50], and from the formative evaluation phase of one of our randomized controlled trials (RCTs) [51]. In this trial, surveys were developed and pretested for implementation with US English speakers with low health literacy across a large geographic area. This included formative evaluation (key informant interviews and focus groups, exploring offline data capture) and, later, process evaluation steps (tracking participant engagement) to determine how and under what circumstances various mHealth strategies increased project engagement. Platforms that were considered for survey implementation included: (1) use of a vendor to manage all online surveys with sophisticated programming and support, (2) use of a commercial product such as Survey Monkey, and (3) use of REDCap. REDCap was chosen as the mHealth platform because (1) it is HIPAA protected; (2) there is no cost to the project; (3) programming support is available; and (4)respondents who are located anywhere geographically can access it using any common device such as personal computers, laptops, tablets, or mobile phones with app capabilities.

The process of enabling the system began with survey instrument development on the REDCap website platform, and then offline module approval was obtained from the US institution’s REDCap administrator. This approval is in the form of a Quick Response Code (QR Code), which contains a unique identifier for each specific requester and is necessary for installation of the survey into the offline application. This QR Code is provided for each staff member collecting data in the field, and each QR code matches with each username. The ability to scan this QR code with the mobile device enhances the project’s ability to collect secure data in the field, including the facilitation of later data collection when offline. Failure to match in this way, however, results in a substantial data storage complication (a “metadata conflict”), whereby the username and the QR code do not match and data collection from that username cannot occur and/or data may be compromised. This code is scanned to each mobile device where the username is used (eg, mobile phones and tablets).

Where online connections are possible, the mobile application provides direct data entry into the REDCap environment on the institution’s server. In LMICs or other low-resource settings where there are limited wireless (WiFi) or cellular connections to ensure predictable online access, the mobile application provides the flexibility for offline data collection. This expanded ability to use digital devices to collect data also limits the need for paper surveys (except as a backup) and the risk of losing data. The portability is also maximized by the ability to be used on iOS and Android platforms (including tablets and mobile phones). With both online and offline options, there is capability for direct data entry, which reduces the time needed for further data management and analysis. Finally, although matching each portable device to unique users requires advanced planning and start-up time, registering each username individually allows for superior tracking of data collection by each data collector.

#### Survey 2: Maternal Health Information Communication Technology

A second survey development and implementation project continued to provide lessons learned for overall project methodologies (ie, formative evaluation for Survey 1 as well as Survey 2) regarding the refinement of the development of both the online and offline survey procedures. The Survey 2 goals in this second project (“MundoComm” based in Costa Rica and the Dominican Republic) include conducting qualitative and quantitative assessments of multi-level determinants of maternal health behaviors that can potentially be addressed through technological innovation, and identifying electronic readiness (e-readiness), defined as use and acceptability of current information communication technology resources (such as social media, texting, as well as earlier technologies such as radio) to guide project development.

During a week-long “short course” training in Costa Rica with teams from the Dominican Republic, Honduras, and Costa Rica, experiences with the offline digital tablet (iPad) demonstrations in vivo revealed new challenges, primarily that systematic upgrades to the REDCap platform at our US-based institution caused immediate problems in the current version accessible on the digital tablet. It was determined that additional or back-up strategies to save all data collected since the last upload/sync will need to be developed. Also, explaining the multi-step processes to gain initial online access at the US-based institution, the steps needed to ensure current access, survey administration, data syncing and trouble-shooting, were all challenging for both instructors and trainees.

## Results

### Survey 1

Selected survey results from Survey 1 with 32 respondents interviewed face-to-face in Puerto Rico with the offline REDCap mobile application (see below) can be found in Table 1. These pilot data, successful collection of which provided evaluation feedback regarding the utility of the offline REDCap survey process, included common demographic information (gender, age, race, ethnicity, education, religion), demographic information of interest to the project (general health status, economic indicators such as owning one’s own house and/or vehicle), and process information on respondents’ perceptions of the length of the survey, and understandability, which aided the project team in refinement of the instrument both for content and for implementation.

**Table 1:** Example variables demonstrating the feasibility of and feedback regarding offline data collection process (N=32).

Variable		% (n/N)
Gender		
	Males	31% (10/32)
	Females	69% (22/32)
Age in years		
	Mean (SD)	35.69 (12.39)
	Range	18-61
Ethnicity		
	Hispanic	100% (29/29)
Race^a^		
	White	80% (24/30)
	Black	13% (4/30)
	Other	27% (8/30)
Education		
	Less than high school	7% (2/29)
	High school	45% (13/29)
	Some college	21% (6/29)
	College	24% (7/29)
	Advanced	3% (1/29)
Religion		
	Christian	97% (28/29)
	Prefer not to answer	3% (1/29)
General health		
	Excellent	17% (5/29)
	Very good	31% (9/29)
	Good	28% (8/29)
	Average	21% (6/29)
	Poor	3% (1/29)
Own your own house		48% (14/29)
Own your own vehicle		59% (17/29)
Understood survey?		
	Totally disagree	28% (8/29)
	Agree	55% (16/29)
	Totally agree	17% (5/29)
Was the survey clear/ simple?		
	Totally disagree	24% (7/29)
	Disagree	10% (3/29)
	Neither agree or disagree	7% (2/29)
	Agree	45% (13/29)
	Totally agree	14% (4/29)

^a^Some selected more than one race.

Feedback from data collectors during their 3-hour training workshop and before the pilot implementation included concerns that the survey was too long and that respondents would likely provide socially desirable responses to speed up the process. Changes to the instrument after pilot implementation resulted from additional feedback, including content related issues (item wording, skip patterns), as well as formatting and navigation issues related to the mobile offline version of the instrument. Data collectors reported that some aspects of the survey (eg, Likert scales) were too difficult for some respondents to understand. More than two thirds of the respondents with whom the survey was piloted, however, indicated that they felt the survey was understandable.

### Survey 2

**Figure 1:**
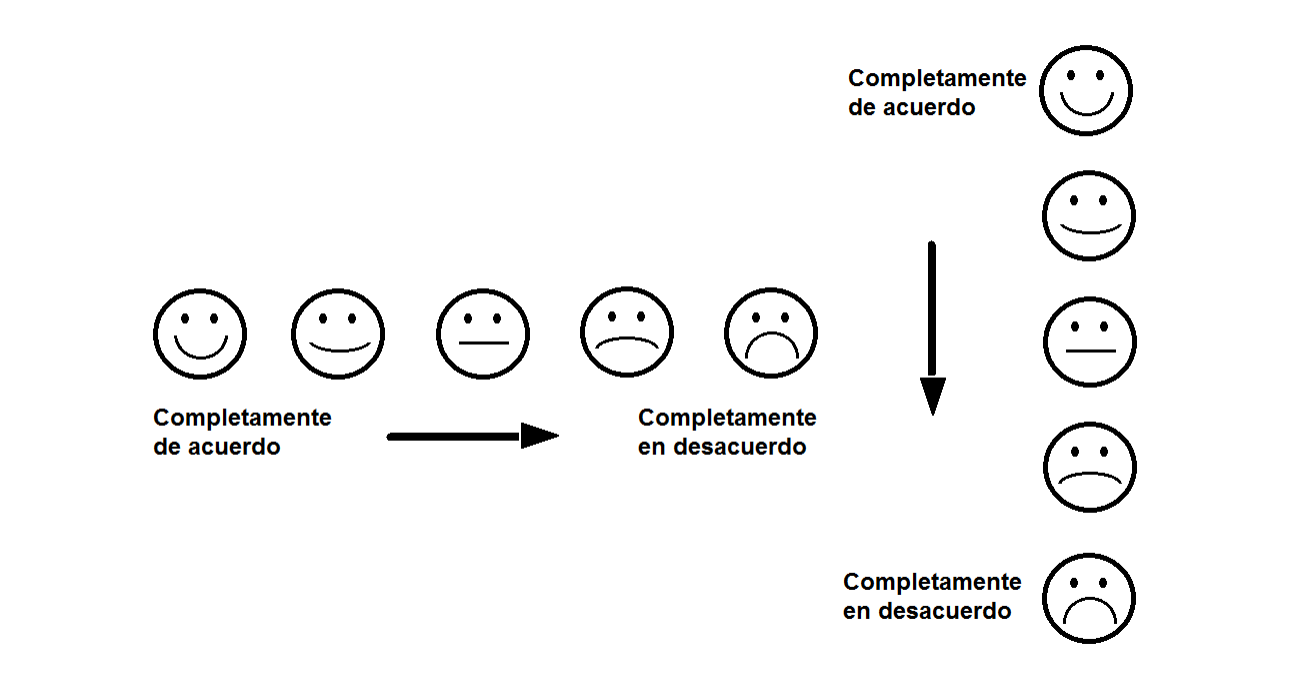
Pictoral ranking scale options.

Pre-testing feedback on Survey 2, which was presented to 16 trainees at a week-long short course in Costa Rica (see above), included similar themes as those identified in feedback regarding Survey 1. Primarily, the participants reported that the survey was too long, item formats such as those presented as Likert Scales or similar ranking tasks, and layout were likely to be confusing to the target population of women in their maternal and child health research projects in their LMICs (Costa Rica, Dominican Republic, and Honduras). An interesting finding was that the Honduran team (n=4) felt strongly that the proposed layouts for the Likert scale items using pictures as aids should not go from left-to-right, as is common in US survey instruments (and even those commonly used in other LMICs), but rather from “top to bottom” (Figure 1).

All qualitative feedback from participants, as well as from LMIC partners and project personnel, will continue to be synthesized with process feedback from Survey 1 (the survey instrument as implemented in the medium of offline data collection), and content feedback (where overlap exists) to edit and pre-test subsequent iterations of Survey 2.

### mHealth Survey Development Challenges

A number of challenges to the development of our mHealth technologies for both online and offline survey data collection were noted. Implications and potential solutions were derived from a review of field notes, feedback from survey respondents and key informant interviewees, feedback from project partners in Puerto Rico and Costa Rica, and project team planning meetings (Table 2). A data collector’s individually assigned username can only used by that data collector, for both online and offline data collection. Therefore, it was decided that it is best to assign only one device to be used by each data collector. If more than one data collector will use a specific device, or more than one person will use a unique username (both possible but not advisable), then additional steps are needed to allow for this expanded functionality and additional training and quality assurance steps would need to be added.

**Table 2:** mHealth survey development challenges.

Problem	Implication	Potential solution
Institutional review board (IRB) approval process	Multiple IRBs and delays were challenges to both initial drafts of methods, and updated methods and instruments	Earlier engagement with IRBs and allotment of more time for ethical reviews and approvals
		Obtain ethical reviews from a centralized IRB process
Unique user identifications	Limits data collection options, such as one device for one collector only	Allow more users access to a single device (eg, a “generic” user ID log in)
Securing device	Although securing the device is meant as a security measure, all data are lost when the device sleeps, is turned off, or the application is accidentally closed	This security measure could be removed, or the device configured to never sleep^a^
Survey is too long	Respondents and collectors felt the instrument is too long and responses won't be valid	The survey will be revised for length and complexity of item response formatting (eg, simpler Likert scales)

^a^This remains a key training and quality improvement issue.

In addition, each data collector must “secure” the instrument prior to data collection (a step in the process of setting up each mobile device). Otherwise, when the device goes into “sleep mode” or the screen is accidentally turned off (screen), there is a risk of losing data already collected. Similarly, failure to match each device with appropriate permission codes can result in a “metadata error” and loss of collected data when attempting to sync the data. Clear protocols are needed to ensure that these steps are taken by all data collectors. For example, project procedures and checklists can be affixed to mobile devices (and included as a “read me” file after the device is activated) to remind data collectors and project staff of important details.

As can be expected in survey development between countries and cultures, several issues were identified, such as challenges with understanding certain survey item formats (eg, Likert scales), and feedback that the survey is “too long”. Finally, the mTurk Spanish version is still in development, and has therefore not been validated. Ongoing process evaluation will continue to identify and resolve these and other survey research implementation issues.

## Discussion

### Principal Findings

Electronic data capture is increasingly common in low and middle income resource settings because of the efficiencies introduced, speed, and convenience. Maximizing these benefits in survey research in settings with limited technological infrastructure and low resources means identifying low-cost mobile strategies with easy user interfaces, yet provide secure and efficient data management. Using a store-and-forward approach, where offline data capture can maximize mobility and reach, offers new successful methods and procedures for survey research.

In our early fieldwork for related public health initiatives, we have tested several electronic data capture modalities in a range of settings, including rural Puerto Rico, and with Latin American public health professionals in Costa Rica, including public health teams from other LMICs (Honduras and the Dominican Republic). This experience has generated several procedural and methodological lessons learned and insights for ongoing project development in our current and future projects in LMICs and other regions with low resources.

### Lessons Learned

#### Lesson 1

While all cross-cultural research projects involving human subjects in multiple countries have expected multi-tiered ethical review processes [28], the complexities involved in newer digital data capturing methodologies mean that even more advanced planning and time will be needed for IRB approvals.

#### Lesson 2

In addition to testing the content of survey instruments and their items, extra time will be needed for testing the use of novel mHealth technologies (both hardware and software), including formative and process evaluation feedback, a consideration which has been noted in other mHealth development initiatives [51,52].

#### Lesson 3

It is important to incorporate the descriptions of usability and steps involved with mHealth technologies into the training of project staff and staff from the target regions. Communication with and input from project staff, partner staff, and research participants should be incorporated throughout the development, implementation, and feedback processes such as pre-testing, pilot testing, and early fieldwork [52].

#### Lesson 4

It is critical to be flexible and change methods accordingly, including both the content and the users’ experience. mHealth technologies influence and are influenced by the content and structure of the survey instrument and associated methods, so it is important to continue to adapt study methods based on this formative and process evaluation feedback [51-54].

### Limitations

Our study has several limitations. First, our experience is limited to the sites as described, so generalizability is limited. Generalizability is further limited by the fact that with research in LMICs with the United States as a partner, there are typically specific requirements and additional regulatory considerations that add time and complexity to a study (eg, multiple ethical reviews, requirement for a partner with an FWA).

Second, mHealth strategies (hardware, software, research infrastructures, open source vs proprietary) used in the present research were not compared to other mHealth options a priori to select resources that would best meet project objectives. Such comparisons are often crucial for conducting mHealth research, especially in LMICs, in order to more fully protect the fidelity of the research. Additionally, the software and hardware described in the present study may not be available or optimal for LMICs. Future research could proactively conduct comparative effectiveness studies of similar methodologies to meet such needs.

Third, these are preliminary lessons learned at the early stages of two large studies. Insights gained from a retrospective examination of barriers and facilitators to a project’s success may reveal different, even contradictory, conclusions. That said, our experiences are useful and practical and could inform the decisions researchers make around electronic data capture in low resource settings.

### Conclusions

Electronic data capture offers an opportunity for low and middle income regions to participate in large research projects efficiently. Although no a priori comparisons for selection of optimal resources within LMICs were conducted, in the early phases of the project, along with our LMIC partners, we were able to successfully identify and implement feasible practical steps regarding the preparation of both software (online applications and services) and hardware (mobile devices) in advance of implementation of our large survey research initiatives. Survey development successfully incorporated both content development (with pre-testing and translation), and platform development for implementation (using REDCap for both online and offline implementation). Advantages and disadvantages to device and software choices, workflows, and user interfaces need to be weighed as part of necessary formative evaluation of the project [50]. These practical details also impact (and are impacted by) IRB issues, training details, and data management procedures.

There is growing evidence that mHealth strategies remove health care barriers in low and middle resource settings, so the dissemination of evidence-based mHealth methodologies is critical [53,54]. Immediate next steps will include full implementation of mHealth driven surveys in multiple LMIC and other low-resource settings, with plans for ongoing process evaluation to guide methodological improvements as needed. Future research in this area could focus upon further qualitative research to inform improvement and implementation [52], usability, privacy and confidentiality issues, best practices for training, and survey implementation fidelity.

## Abbreviations

AIT: Academic Information Technology
CTS: Clinical and Translational Science Institute
FWA: Federal Wide Assurance
HIPAA: Health Insurance Portability and Accountability Act
IRB: institutional review bard
LMIC: low and middle income countries
mTurk: Mechanical Turk
mHealth: mobile health
NIH: National Institutes of Health
OHRP: Office for Human Research Protections
PROTECT: Puerto Rico Testsite for Exploring Contamination Threats
REDCap: Research Electronic Data Capture
QR Code: Quick Response Code
